# Indirubin and Indirubin Derivatives for Counteracting Proliferative Diseases

**DOI:** 10.1155/2015/654098

**Published:** 2015-09-17

**Authors:** Tina Blažević, Elke H. Heiss, Atanas G. Atanasov, Johannes M. Breuss, Verena M. Dirsch, Pavel Uhrin

**Affiliations:** ^1^Department of Pharmacognosy, University of Vienna, 1090 Vienna, Austria; ^2^Department of Vascular Biology and Thrombosis Research, Center for Physiology and Pharmacology, Medical University of Vienna, 1090 Vienna, Austria

## Abstract

Indirubin is the active component of Danggui Longhui Wan, a traditional Chinese medicine formulation. The encouraging clinical results from the 1980s obtained in chronic myelocytic leukemia patients treated with indirubin stimulated numerous studies on this compound. These investigations explored the use of indirubin in different types of cancer and reported the synthesis of novel derivatives with improved chemical and pharmacokinetic properties. In this paper, we review the impressive progress that has been made in elucidating the mechanistic understanding of how indirubin and its derivatives affect physiological and pathophysiological processes, mainly by inhibition of cell proliferation and induction of cell death. Furthermore, we survey the therapeutic use of these compounds in combating proliferative diseases such as cancer, restenosis, and psoriasis.

## 1. Introduction

Historically, natural products have been successfully used in the management of a large number of human diseases [1]. Natural products may also serve as a basis for synthesis of derivatives aiming to reduce toxic side effects, to improve their pharmacokinetic properties, and to increase their efficacy [1–3]. The molecular targets and mechanisms of these substances in physiology or pathophysiology have been elucidated only in the last decades.

Indirubin is the active ingredient of Danggui Longhui Wan, a traditional Chinese medicine containing plants such as* Indigofera tinctoria* L. and* Isatis tinctoria* L. The interest in clinical use of indirubin was evoked in the 1980s in China when medical doctors together with scientists started testing its clinical use for treatment of chronic myelocytic leukemia (CML), a slowly progressive disease characterized by the overproduction of granulocytes [4–7]. Over 50% of the treated CML patients exhibited partial or complete remission [6, 8, 9], similar to the standard treatment using the cytostatic agent busulfan [8]. Indirubin toxicity was low and the side effects experienced by about half of the participants comprised mild abdominal pain, diarrhea, and nausea [7]. In three cases reversible pulmonary arterial hypertension and cardiac insufficiency were reported [10].

These encouraging results with CML stimulated researchers to explore the use of indirubin and its novel derivatives in other types of cancer as well as other diseases [11–14]. Here, we survey the outcome of these studies in combating proliferative diseases, such as cancer, pathological angiogenesis, restenosis, and psoriasis, and discuss the underlining mechanisms regarding how indirubin influences cellular signaling. In the text, we refer to natural indirubin as well as its chemical derivatives as indirubins.

## 2. Synthetic Indirubin Derivatives

Indirubin, a stable red isomer of the blue indigo, is chemically a 3,2′-bisindole. In many laboratories the structure of indirubin was employed as a skeleton for the synthesis of new derivatives, with improved chemical and pharmacological properties such as solubility and absorption. The antiproliferative effects of indirubins were mostly attributed to the inhibition of cell cycle-related kinases, like cyclin-dependent kinases (CDKs) and glycogen synthase kinase-3*β* (GSK-3*β*), and later on to other target proteins, as described further in the text. New indirubin derivatives, in addition to increased antiproliferative properties, served as important tools in revealing specific targets that mediate their cellular effects.

The first classes of synthesized indirubin-based compounds included alkylated, halogenated, and N- and O-substituted derivatives. Among these, N-ethyl-indirubin, 5-halogen-indirubins, and N-methylisoindigotin (meisoindigo) demonstrated much higher antitumor potency in animal models than natural indirubin [15–17]. Indirubin-3′-monoxime (a substance with numerous synonyms such as indirubin-3′-oxime, indirubin-3-oxime, indirubin-3-monoxime, indirubinoxime, and indirubin-3′-monoxime) was synthesized in 1996 by Li and colleagues [18] and was later found to inhibit CDKs, a major target of indirubins, with higher potency when compared to the parent compound [9]. To date, indirubin-3′-monoxime has become one of the most studied synthetic indirubin derivatives. This substance similar to some other newly synthesized indirubins (5-iodoindirubin-3′-monoxime, indirubin-5-sulfonic acid, indirubin-5-sulfonamide, and 5-halogenoindirubins) inhibits efficiently, at nanomolar range, another indirubin target, the GSK-3*β* [19]. Next, using cocrystal structures and modeling approaches, novel and more potent CDK and GSK-3*β* inhibitors such as 6-bromoindirubin were discovered [33]. Thereafter, 6-bromoindirubin-3′-oxime having higher potency towards GSK-3*β* was developed [20]. Additionally, novel 3′-substituted 7-halogenoindirubins lacking the inhibitory effects towards CDKs and GSK-3*β* were generated, which nevertheless still induced cell death in a diversity of human tumor cell lines [21]. This allowed researchers to point to the importance of other mechanisms mediating indirubin effects [21]. The synthesis and characterization of several other derivatives, such as E564, E728, and E804, demonstrated the inhibitory effect of these compounds towards signal transducers and activators of transcription 3 (STAT3) signaling, thereby contributing to the induction of apoptosis in human cancer cells [22]. STATs are transcription factors that transmit signals from membrane receptors to the nucleus, where they promote the transcription of their target genes or play an important role in regulating cell cycle progression and in apoptosis [34, 35].

The study on the novel indirubin derivatives 5-nitro-indirubinoxime, 5-fluoro-indirubinoxime, and 5-trimethylacetamino-indirubinoxime (originally [23] denoted incorrectly as substituted at the five-prime position on the indirubin molecule) demonstrated their antitumor activity* in vitro* and in several animal models for cancer [23–27], while 5-nitro-indirubinoxime additionally exhibited anti-inflammatory properties in human vascular endothelial cells [28]. In comparison with other known indirubins, 7-azaindirubin bearing a heterocyclic nitrogen atom at position C7 as well as its 3′-oxime derivative shows reduced kinase inhibitory activity but nevertheless demonstrates high antiproliferative activity [29]. Newly synthesized (di)azaindirubins exhibit a highly selective inhibitory activity against casein kinase 2, as revealed by testing of a panel of more than 200 protein kinases [36]. PHII-7 became a promising potent derivative of indirubin with cytotoxic effects in multidrug resistant cells, which represent a major obstacle for chemotherapy in many human malignancies [37]. Indirubins carrying a 5′-carboxylate moiety (such as compound 7-bromo-5′-carboxyindirubin-3′-oxime) yielded a novel inverse binding mode with improved selectivity for DYRK kinases that are implicated in alternative pre-mRNA splicing and in pathologies associated with neurons, including Alzheimer's disease and Down syndrome [30]. Finally, 5-diphenylacetamido-indirubin-3′-oxime was described as a novel mitochondria-targeting agent with antileukemic activities [31].

Due to the planarity of structure, the developed hydrogen bonds and hydrophobic *π*-interactions and the rigid crystal structure indirubins generally suffer from low water solubility [38]. Achieving an increased, though not excessive, water solubility, without loss of bioactivity, represents a major goal in the synthesis of new indirubins [20]. To this end, attempts to intervene in the scaffold of indirubin (e.g., the conversion of 3′-carboxyl group to an oxime) and to destroy the planarity of indirubins have been made [39]. Alternatively, chemical modifications by adding polar hydrophilic groups to the scaffold were accomplished, as in the case of novel analogues of 6-bromo-indirubin-3′-oxime [40]. The latter substances were then successfully applied to alter circadian rhythm in cell cultures [40]. Lately, indirubin-5-carboxamides, a novel class of indirubins carrying amide substituents with basic centers, were generated. Here quaternization or protonation of the alkylamino substituents improved their solubility, also without loss of bioactivity [41]. The recently developed 5′-OH-5-nitro-indirubin oxime (AGM130) had improved solubility compared to indirubin and was effectively applied to induce apoptosis of CML cells that were resistant to imatinib, a blocker of the pathological BCR/ABL fusion protein [32]. Additionally, extensive series of more soluble halogenated analogues have been generated, as reviewed elsewhere [13, 42]. Also the structure of meisoindigo has been modified and the derivatives have been tested in a panel of cancer cells [43]. The substance (E)-1-(2-(4-methylpiperazin-1-yl)ethyl)-[3,3′-biindolinylidene]-2,2′-dione (5-4) was at least 40 times better soluble than meisoindigo and exhibited little or no tendency to aggregate in solution and was capable of significantly extending the lifespans of animals in a K562 tumor xenografts model [43].

Besides the attempts to increase solubility of the indirubins, studies aiming to increase their* in vivo* bioavailability have been performed. These include, for example, the establishment of supersaturatable self-microemulsifying drug delivery system (S-SMEDDS) that improved indirubin's release* in vitro* and its bioavailability in rats when orally applied [44]. In another study use of a self-nanoemulsifying drug delivery system (SNEDDS) increased oral bioavailability of E804 by ~980% when compared with the aqueous suspension [45].

Typical features of some of these above-mentioned indirubins as well as their structures are depicted in Table 1.

## 3. Proteins and Signaling Pathways Targeted by Indirubins

The intensive research during the several last decades on indirubins revealed that these substances interact with different proteins and influence a number of signaling pathways. The main effects of indirubins include the inhibition of cell proliferation as well as induction of cell differentiation and cell death, as surveyed below.

### 3.1. Indirubin-Mediated Inhibition of Cell Proliferation and Induction of Differentiation

The suppression of malignant transformation in CML patients by indirubin, reported in the 1980s, was originally attributed to DNA intercalation [17, 46] as well as interference with microtubule polymerization [17]. Only later, the potent inhibition of CDKs and of GSK-3*β* was found to contribute mainly to the antiproliferative effects of indirubins [9, 19]. Besides these effects, indirubin from Realgar-Indigo naturalis formula used for APL patient treatment caused intensified ubiquitination/degradation of the promyelocytic leukemia- (PML-) retinoic acid receptor alpha (RARalpha) oncoprotein [47].

CDKs are very important regulators of the eukaryotic cell cycle [48]. Identification of CDKs as specific molecular targets of indirubins was reported by the group of Meijer [9]. Their work discovered the cocrystal structure of a complex of CDK2 and indirubin-3′-monoxime, thereby demonstrating indirubin-3′-monoxime interaction with the ATP-binding site of CDK2. In a later study, indirubin-3′-monoxime potently inhibited cell proliferation of a mammary carcinoma cell line (MCF-7), by arresting the cells in the G2/M phase of the cell cycle, possibly by inhibiting CDK1 [49]. Furthermore, indirubin-3′-oxime suppresses in a CDK2-dependent manner abnormal centriole duplication induced by the human papillomavirus in a human osteosarcoma cell line [50]. The compound also increases the level of the CDK inhibitor p27^Kip1^ in a human neuroblastoma cell line (LA-N-1) and reduced the expression of CDK2 and cyclin E, leading to cell cycle arrest at the G0/G1 phase [51].

Cell cycle-inhibitory (cytostatic) activity of indirubins was additionally attributed to the inhibition of GSK-3*β* [19], a regulatory serine/threonine kinase related to CDKs. GSK-3*β* exhibits a plethora of cellular effects and is a therapeutic target for the treatment of cancer, chronic inflammation, type II diabetes, and other diseases [52, 53]. Such inhibition by 6-bromoindirubin acetoxime was important not only for suppression of migration of adult gliomas [54] but also for inhibiting migration of pediatric glioma, by instigating cytoskeletal rearrangement [55].

Another mechanism for how indirubins contribute to their cytostatic effects is the interaction with the aryl hydrocarbon receptor (AhR) [56]. AhR plays an important role in the cellular metabolism of xenobiotics, and AhR signaling is considered as a promising drug target, particularly for cancer, inflammation, and autoimmune diseases [57]. Indirubin and indigo bind to AhR with high affinities [58]. These compounds are present in normal human urine at 0.2 nM concentration and at such physiological levels they may activate AhR-mediated signaling mechanisms* in vivo* [59]. The kinase-independent cytostatic effect of indirubins was demonstrated using 1-methyl-indirubins, a subfamily of kinase-inactive indirubins, and involved the AhR-mediated expression of a potent CDK2 inhibitor p27^Kip1^ [56]. In another study, overexpression of the AhR in the absence of exogenous ligands was found to rapidly disrupt centriole duplication control in breast cancer cells [60]. Nonetheless, the AhR agonist, indirubin-3′-oxime, and a kinase-inactive inhibitor, 1-methyl-indirubin-3′-oxime, still significantly reduce centriole overduplication stimulated by ectopic AhR expression [60]. The role of indirubins in AhR signaling seems to be complex, as indirubin activated AhR in human HaCaT and HepG2 cells with significantly higher, although still transient, potency as compared with dioxin, the prototypical AhR ligand [61].

Finally, indirubins may induce cell differentiation or inhibit cellular proliferation by suppression of protooncogenes or by interference with growth factor signaling. For example, the indirubin derivative meisoindigo was shown in the 1990s to induce differentiation of ML-1 human myeloblastic leukemia cells and to downregulate c-myb gene expression [62]. Indirubin-3′-monoxime suppresses the autophosphorylation of fibroblast growth factor receptor 1 (FGFR1) and, by blocking receptor-mediated cell signaling, also inhibits proliferation of NIH/3T3 fibroblasts [63]. Indirubin-3′-monoxime further potently suppresses Notch1 signaling in a manner that is dependent on GSK-3*β* but independent of proteosomal degradation [64]. The compound 5-nitro-indirubinoxime (denoted incorrectly as substituted at the five-prime position on the indirubin molecule) inhibits the beta1 integrin/focal adhesion kinase/Akt pathway and thereby decreases the metastatic ability of human head and neck cancer cells [26]. Indirubin-3′-monoxime in human leukemic KBM-5 cells suppresses tumor necrosis factor- (TNF-) *α*-induced activation of NF-*κ*B, a central transcription factor involved in the regulation of various inflammatory genes as well as antiapoptotic genes [65, 66]. Although the roles of NF-*κ*B in different types of cancer are complex [67], these data indicate that the antiproliferative and the anti-inflammatory activity previously assigned to indirubin [28] may be mediated in part through the suppression of the NF-*κ*B pathway.

### 3.2. Indirubin-Mediated Induction of Cell Death

Additional important hallmarks of the indirubin-related effects, which include induction of proapoptotic genes, interference with mitochondrial function, and STAT signaling leading to cell death, are surveyed below.

**Figure 1:**
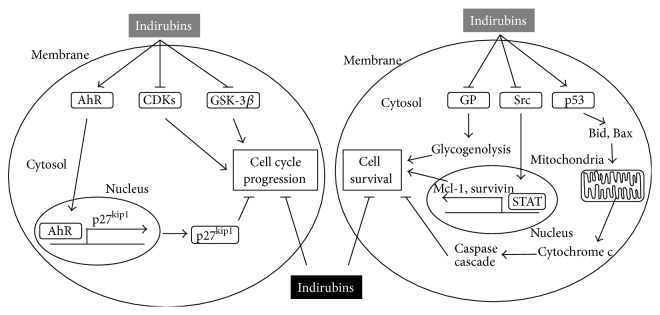
Cellular effects mediated by indirubins. By direct inhibition of CDKs and GSK-3*β* and by the activation of AhR-mediated upregulation of cell cycle inhibitor p27^Kip1^, indirubins inhibit cell cycle progression. They also suppress cell survival by inducing caspase-mediated apoptosis upon interfering with mitochondrial function and affecting Src and STAT-mediated expression of cell survival proteins. Furthermore, by inhibiting GP indirubins suppress glycogenolysis, thus hampering the glucose supply of cancer cells. These effects lead to inhibition of cell cycle progression and induction of cell death (→ activation/release; ⊥ inhibition).

Indirubin-5-nitro-3′-monoxime in human lung cancer cells induces apoptosis via p53- and mitochondria-dependent pathways [68]. The compound additionally stimulates cell cycle arrest as well as apoptosis, as shown with carcinoma cells. This process was instigated after the release of cytochrome c and the activation of caspases. Also, indirubin-3′-monoxime causes apoptosis in human cervical, hepatoma, and colon cancer cells by inducing proapoptotic Bcl-2 family members Bid and Bax [69], as depicted in Figure 1. Indirubin-3′-oxime (synonym of indirubin-3′-monoxime) further alters mitochondrial function in human neuroblastoma cells by curtailing the mitochondrial membrane potential and elevating the levels of reactive oxygen species [51]. Moreover, this substance induces apoptotic and autophagic death in acute lymphoblastic as well as chronic myelogenous human leukemia cells, by a mechanism possibly involving caspase-3 [70].

An additional indirubin derivative, 5′-methoxyindirubin, induces apoptosis in human neuroblastoma cells [71]. Treatment of melanoma cells with 8-Rha-*β*-indirubin enhances their sensitivity for death ligands, overcoming their resistance to two important factors, namely, TNF-*α*-related apoptosis-inducing ligand (TRAIL) and CD95 agonists [72]. A similar drug-potentiation effect was observed for the novel indirubin derivative PHII-7 that increased adriamycin cytotoxicity (via inhibiting P-glycoprotein expression) and cell apoptosis in MCF-7/ADR human breast cancer cells [37]. Application of 6-bromo-indirubin-3′-oxime effectively abrogates cellular growth and induces apoptosis in both breast cancer and bladder carcinoma cell lines and prevents tumor cell resistance towards TRAIL [73]. Finally, 5-diphenylacetamido-indirubin-3′-oxime was recently described as a novel mitochondria-targeting agent with antileukemic activities that is able to increase the opening of mitochondrial permeability transition pores [31].

In many reported cases, indirubin-induced apoptosis was caused by an interference with STAT signaling. For example, indirubin derivatives E564, E728, and E804 potently block constitutive STAT3 signaling in human breast and prostate cancer cells [22]. The compound E804, in addition, directly inhibits Src, a kinase acting upstream STAT proteins, and suppresses the expression of antiapoptotic proteins Mcl-1 and survivin [22]. Importantly, a subtoxic concentration of E804 resensitized cancer treatment-resistant cells (MV4-11-R) by inhibiting STAT1, STAT3, and STAT5 signaling and by abolishing survivin expression [74]. In human K562 chronic myelogenous leukemia cells, E804 potently suppresses STAT5 signaling and induces apoptosis [75]. E738 works as a novel dual inhibitor of STAT-activating kinases, including Janus kinases (JAKs) and Src family kinases (SFKs), thereby inducing apoptosis in human pancreatic cancer cells [76]. In human melanoma cells, 6-bromoindirubin-3′-oxime inhibits JAK and STAT3 signaling [77] and 7-bromoindirubin (designated as MLS-2438) inhibits STAT3 and Akt signaling, with both derivatives inducing apoptosis [78].

Besides the ability to induce apoptosis, indirubins also induce necrosis. Specifically, indirubin-3′-monoxime causes necrosis in human breast carcinoma cell line [79] and 7-bromoindirubin-3′-oxime instigates necrosis in human THP-1 macrophages and in a human mesothelioma cell line [80].

Finally, indirubin-5-sulphonate allosterically inhibits glycogen phosphorylase (GP) [81]. This effect might be important for the depletion of glucose in cancer cells, thereby hampering their survival.

The main pathways of indirubin-mediated effects on proliferation and cell death are outlined in Figure 1.

## 4. Indirubins Counteracting Proliferative Diseases

In addition to the* in vitro* effects outlined above, indirubins were tested and their potency was shown in many preclinical models. These studies clearly showed the potential therapeutic use of indirubins for counteracting proliferative diseases, such as cancer, pathological angiogenesis, restenosis, and psoriasis, as reviewed in the following sections.

## 5. Indirubins Thwart Cancer and Pathological Angiogenesis

One of the first animal studies pointing to an efficacy of indirubins in the treatment of cancer involved the application of various indirubins to skin xenografts. Specifically, direct injection of indirubins into xenografts of RK3E-ras rat kidney epithelial cells harboring the k-ras gene was shown to reduce cancer in male Sprague-Dawley rats [23]. In the aforementioned study, several indirubins such as 5-nitro-indirubinoxime, 5-fluoro-indirubinoxime, and 5-trimethylacetamino-indirubinoxime were injected every other day for 10 days, and this significantly inhibited tumor growth and induced apoptosis [23]. Indirubin-3-monoxime was applied to a murine benzo(*α*)pyrene [B(*α*)P]-induced lung cancer model at a dose of 10 mg/kg for 5 d per week, and this resulted in a reduced adenocarcinoma growth due to apoptosis [82]. Clinically, treatment of a CML patient using imatinib in combination with indirubin and meisoindigo resulted in complete cytogenetic response and the longest record of survival among CML patients in the literature [83]. The isoindigo derivative natura-alpha (N-methyl-Δ3,3′-dihydroindole-2,2′-diketone) demonstrated both* in vitro* and* in vivo* effects. In case of the former, growth of both androgen-dependent (LNCaP) and androgen-independent (LNCaP-AI, PC-3, and DU145) prostate cancer cells was inhibited, whereas the compound inhibited androgen receptor signaling upon coinjection with human prostate cell lines in a xenograft model using nude mice [84]. Importantly, natura-alpha also reduced tumor volume in a patient with hormone-refractory metastatic prostate cancer [84]. Indirubin-3′-monoxime also induced apoptosis in oral cancer cell lines* in vitro*, through activation of cytochrome c and inhibition of a number of factors including focal adhesion kinase, urokinase-type plasminogen inhibitor, and matrix metalloproteinase. When applied topically to mice in an adhesive gel, indirubin-3′-monoxime suppressed oral cancer through the downregulation of survivin [85].

In addition to their ability to suppress primary tumour growth, indirubins also suppress lung tumour metastases, as demonstrated by administration of 6-bromo-indirubin-3′-oxime in a 4T1 mouse model for aggressive breast cancer [86]. These findings were fully consistent with* in vitro* data showing that 6-bromo-indirubin-3′-oxime inhibited adhesion, migration, and invasion of a variety of metastatic cell types. This was achieved by influencing the JAK/STAT3 signaling pathway and inhibiting several kinase cascades involved in the metastasizing of cancer cells. Such competence of indirubins to influence multilaterally several cellular signaling networks and metabolism strongly suggests that indirubins could be used as drugs, either alone or in combination with conventional antineoplastic agents, to overcome multidrug resistance, which represents a major impediment to the effective therapy of many human malignancies [87].

The experimental evidence for the importance of inducing and sustaining angiogenesis, the formation of new blood vessels from existing ones, for tumor growth is compelling [88, 89]. Pathological angiogenesis is a hallmark of cancer as well as various ischaemic and inflammatory diseases [90–92]. Therefore, the ability of indirubins to inhibit angiogenesis, as outlined below, may significantly contribute to the inhibition of tumor growth and metastasis.

The antiangiogenic potential of indirubin-3′-monoxime was discovered* in vivo* in an automated quantitative screening assay using transgenic zebrafish embryos and confirmed* in vitro* by applying tube formation and proliferation in human umbilical vein endothelial cells HUVECs [93]. In a subsequent study, indirubin-3′-monoxime was applied to endothelial cells, which not only influenced proliferation, cell cycle, apoptosis, migration, and tube formation but also acted on AKT, p38, ERK1/2, c-Src, and GSK-3*β* kinases. Indirubin's scaffold was therefore suggested to be promising for the development of novel antiangiogenic drugs [94]. Additionally, the inhibitory effect of the parent compound indirubin on angiogenesis was visualized in transgenic zebrafish embryos and confirmed in endothelial cell cultures [95]. 5-Nitro-indirubinoxime reduced angiogenesis, primarily by inhibiting the expression of vascular endothelial growth factor VEGF [26]. This was shown* in vivo* using the chorioallantoic membrane (CAM) assay in fertilized chicken eggs and in the cornea of mice [26]. In* ex vivo* assays, indirubin-3′-monoxime was also able to inhibit the number of microvessels growing from the aortic rings, whereas in other* in vivo* tests using mice the compound induced neovascularization in matrigel plugs. The molecular mechanisms underlying the antiangiogenic effects of indirubin-3′-monoxime in endothelial cells depended, at least partly, on the downregulation of the activation of VEGFR2 (vascular endothelial growth factor receptor 2) [96, 97]. The* in vivo* inhibitory effect of E804 on angiogenesis and tumor growth was shown in syngenic Balb/c mice that were transplanted subcutaneously with colon cancer cells. Subsequently, the intratumor injections of E804 inhibited vascular growth of the inoculated allografts, as demonstrated by a decrease in both the microvessel density marker CD31 and the proliferative indicator Ki-67, but also by an increase in the apoptosis index [98]. The antiangiogenic effect of E804 was further corroborated* in vitro* by inhibition of proliferation, migration, and tube formation of HUVECs and* in vivo* by monitoring growth factor-induced neovessel formation in the matrigel plug model in mice [99]. These growth factors included VEGF and basic fibroblast growth factor (bFGF). Altogether, these data clearly show the potency of indirubins to suppress cancer growth, in part by inhibiting pathological angiogenesis.

## 6. Indirubins Counteracting Restenosis

Restenosis refers to the thickening of the arterial wall and is characterized not only by increased platelet and immune cell infiltration but also by increased migration and proliferation of vascular smooth muscle cells (VSMCs) into the intimal layer. Restenosis represents a serious clinical problem in patients undergoing balloon angioplasty and stenting and may result in life-threatening vessel occlusions [100, 101]. Studies on VSMC proliferation* in vitro* and experimentally induced neointima formation* in vivo* revealed the inhibitory action of indirubin-3′-monoxime on restenosis. In our previous work, indirubin-3′-monoxime inhibited VSMC proliferation induced by the BB isoform of platelet-derived growth factor (PDGF-BB) by arresting cells in the G0/G1 phase of the cell cycle. Moreover, the indirubin derivative specifically blocked the phosphorylation of STAT3 upon PDGF, interferon-*γ*, and thrombin stimulation [102]. In a murine femoral artery cuff model, indirubin-3′-monoxime prevented neointima formation while reducing both STAT3 phosphorylation and the amount of proliferating Ki67-positive cells. In addition, this compound increased endothelial nitric oxide production, thereby potentially ameliorating endothelial dysfunction [102]. A further study on indirubin-3′-monoxime demonstrated that it inhibits the migration of vascular smooth muscle cells after stimulation with leukotriene or platelet-derived growth factor. Moreover, the compound suppresses leukotriene biosynthesis in monocytes by direct inhibition of 5-lipoxygenase [103]. Oral administration of indirubin-3′-monoxime significantly retarded occlusion in a rat carotid artery-injury model as well as ADP- and collagen-induced platelet aggregation. This effect was due to the suppression of glycoprotein VI-mediated signaling pathways in platelets through blocking of phospholipase C*γ*2 phosphorylation [104]. A further compound, 5-nitro-indirubinoxime (incorrectly designated as modified at the five-prime position), was applied to HUVECs and this inhibited the TNF-*α*-induced anti-inflammatory response and the adhesion of U937 cells by decreasing the expression of the cell adhesion molecules ICAM-1 and VCAM-1 [28]. The above described inhibitory effects of indirubins on VSMC proliferation, platelet aggregation, and activation of endothelial cells showcase the potential use of indirubins, especially that of indirubin-3′-monoxime, as lead compounds for preventing restenosis.

## 7. Indirubins for Treating Psoriasis

Psoriasis is a common inflammatory skin disease, affecting about 2% of the general population in the western world [105]. One of the hallmarks of this disease is an uncontrolled proliferation of keratinocytes, which is accompanied by abnormal differentiation of cells [106, 107]. Mechanistic studies revealed that in psoriatic skin lesions indirubin treatment interferes with the proliferation and differentiation of keratinocytes, underscored by a decrease in proliferation marker PCNA and an increase in the expression of protective involucrin [108]. Indirubin additionally inhibits the activation of epidermal growth factor receptor (EGFR) as well as CDC25B gene expression induced by epidermal growth factor (EGF) [109]. In cultured human keratinocytes and in psoriatic lesions, indirubin also causes upregulation of claudin-1, which functions as major constituent of tight junction complexes that regulate the permeability of epithelia [110]. Application of 5-nitro-indirubinoxime (incorrectly [111] designated as modified at the five-prime position) to mouse skin epidermal cells results in the inhibition of neoplastic cell transformation induced by EGF or 12-O-tetradecanoylphorbol-13-acetate (TPA). Such treatment additionally inhibits a number of activities induced by EGF or TPA, including those of Raf-1, MEK1/2, ERK1/2, JNK, and c-Jun. Also, in a mouse model of skin psoriasis, local application of indirubin and of indigo naturalis was demonstrated to be effective and safe [112]. In a recent study, indigo naturalis treatment was additionally shown to be effective in treating nail psoriasis in humans, a condition that is notoriously difficult to treat and which lacks standardized therapeutic regimens [113].

## 8. Conclusions

The use of indirubin and its derivatives in several clinical settings has been patented, as recently surveyed in another review [38], and holds promise for future therapeutic applications. In addition to the traditional use of indirubin in the treatment of CML, indirubin and its numerous derivatives were shown to be effective in counteracting many proliferative diseases, as demonstrated by cell culture studies and preclinical models. This review highlights the contemporary progress in elucidating the mechanisms of how indirubins influence multiple molecular pathways, leading to cell cycle inhibition and apoptosis. Although the most pronounced effects of indirubin and its derivatives were seen in cancer, treatment of psoriasis and restenosis may also benefit from this group of compounds.

## Figures and Tables

**Table 1:** The core structure of indirubin (R^5^=O) with listed structures and actions of selected synthetic indirubin derivatives. X denotes halogen atoms (Br, I, Cl, and F).

Indirubin derivatives	R^1^	R^2^	R^3^	R^4^	R^5^	R^6^	Main effects/properties	Reference
Core structure of indirubins	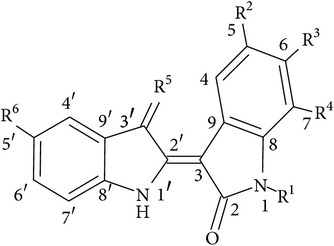							

Indirubin	-H	-H	-H	-H	=O	-H	Antitumor effect	[4–10]

N-Ethyl-indirubin 5-halogenoindirubins N-Methylisoindigotin (meisoindigo)	-CH_2_CH_3_	-H	-H	-H	=O	-H	Higher antitumor potencies compared to indirubin	[15–17]
	-H	-X	-H	-H	=O	-H		
	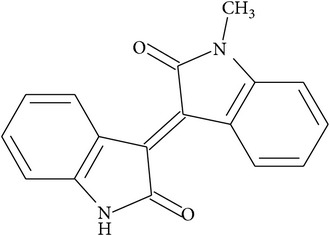							

Indirubin-3′-monoxime	-H	-H	-H	-H	=NOH	-H	Inhibition of CDKs with high potency	[9, 18]

5-Iodoindirubin-3′-monoxime	-H	-I	-H	-H	=NOH	-H	Inhibition of GSK-3*β*	[19]
Indirubin-5-sulfonic acid	-H	-SO_3_H	-H	-H	=O	-H		
Indirubin-5-sulfonamide	-H	-SO_2_NH_2_	-H	-H	=O	-H		
5-Halogenoindirubins	-H	-X	-H	-H	=O	-H		

C6 and C5, C6 halogen substit. of indirubin	-H	-H or -X	-X	-H	=O	-H	More potent CDK and GSK-3*β* inhibitors	[20]

3′-Substituted 7-halogenoindirubins	-H or -CH_3_	-H	-H	-X	=NOH, =NOCH_3_, =NOCOCH_3_ and others	-H	Lacking the inhibitory effects towards CDKs and GSK-3*β* but still inducing cell death	[21]

E564	-H	-H	-H	-H	=NOCH_2_CH_2_OCH_2_CH_2_OH	-H	Inhibitory effect towards STAT3 signaling, contributing to apoptosis in human cancer cells	[22]
E728	-H	-OCH_3_	-H	-H	=NOH	-H		
E804	-H	-H	-H	-H	=NOCH_2_CH_2_CH(OH)CH_2_OH	-H		

5-Fluoro-indirubinoxime	-H	-F	-H	-H	=NOH	-H	Antitumor activity *in vitro* and in several animal models	[23–27]
5-Trimethylacetamino-indirubinoxime	-H	-NH CO^t^Bu	-H	-H	=NOH	-H		
5-Nitro-indirubinoxime	-H	-NO_2_	-H	-H	=NOH	-H	Additional anti-inflammatory properties in HUVECs	[28]

7-Azaindirubin-3′-oxime	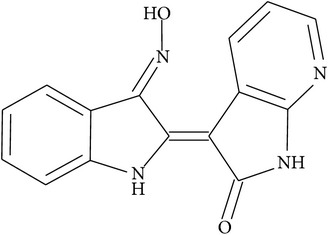						Potent antiproliferative properties in cancer cell lines and inhibition of a series of kinases	[29]

7-Bromo-5′-carboxyindirubin-3′-oxime	-H	-H	-H	-Br	=NOH	-COOH	Novel inverse binding mode, with improved selectivity for DYRK kinases	[30]

5-Diphenylacetamido-indirubin-3′-oxime	-H	-NHCOC H(C_6_H_5_)_2_	-H	-H	=NOH	-H	Novel mitochondria-targeting agent with antileukemic activity	[31]

5′-OH-5-nitro-indirubin oxime (AGM130)	-H	-NO_2_	-H	-H	=NOH	-OH	Improved solubility compared to indirubin and effective induction of apoptosis of imatinib-resistant CML cells	[32]

## The Key Abbreviations Used

AhR: Aryl hydrocarbon receptor
bFGF: Basic fibroblast growth factor
CDK: Cyclin-dependent kinase
CML: Chronic myelocytic leukemia
eNO: Endothelial nitric oxide
EGF: Epidermal growth factor
EGFR: Epidermal growth factor receptor (EGFR)
FGFR1: Fibroblast growth factor receptor 1
GP: Glycogen phosphorylase
GSK-3*β*: Glycogen synthase kinase-3*β*
HUVECs: Human umbilical vein endothelial cells
ICAM-1: Intercellular adhesion molecule 1
PDGF: Platelet-derived growth factor
STAT: Signal transducers and activators of transcription
TNF-*α*: Tumor necrosis factor-*α*
TPA: 12-O-Tetradecanoylphorbol-13-acetate
TRAIL: TNF-*α*-related apoptosis-inducing factor ligand
VCAM-1: Vascular cell adhesion molecule 1
VEGF: Vascular endothelial growth factor
VEGFR2: Vascular endothelial growth factor receptor 2
VSMC: Vascular smooth muscle cell.
